# The composite phenotype analysis identifies potential concerted responses of physiological systems to high altitude exposure

**DOI:** 10.1093/nsr/nwad053

**Published:** 2023-03-01

**Authors:** Yi Li, Meng Hao, Zixin Hu, Yanyun Ma, Kun Wang, Xiaoyu Liu, Xianhong Yin, Menghan Zhang, Yi Wang, Meng Liang, Yuan Guo, Lei Bao, Shixuan Zhang, Shiguan Le, Chenyuan Wu, Dayan Sun, Yang Wei, Fei Wu, Rui Zhang, Lingxian Zhu, Hui Zhang, Shuai Jiang, Xingdong Chen, Xiaofeng Wang, Yao Zhang, Longli Kang, Wenyuan Duan, Bin Qiao, Jiucun Wang, Li Jin

**Affiliations:** State Key Laboratory of Genetic Engineering, Collaborative Innovation Center for Genetics and Development, School of Life Sciences and Human Phenome Institute, Fudan University, China; Institute for Six-sector Economy, Fudan University, China; International Human Phenome Institutes, China; Ministry of Education Key Laboratory of Contemporary Anthropology, Department of Anthropology and Human Genetics, School of Life Sciences, Fudan University, China; Greater Bay Area Institute of Precision Medicine (Guangzhou), School of Life Sciences, Fudan University, China; Ministry of Education Key Laboratory of Contemporary Anthropology, Department of Anthropology and Human Genetics, School of Life Sciences, Fudan University, China; Artificial Intelligence Innovation and Incubation Institute, Fudan University, China; Ministry of Education Key Laboratory of Contemporary Anthropology, Department of Anthropology and Human Genetics, School of Life Sciences, Fudan University, China; Institute for Six-sector Economy, Fudan University, China; Ministry of Education Key Laboratory of Contemporary Anthropology, Department of Anthropology and Human Genetics, School of Life Sciences, Fudan University, China; State Key Laboratory of Genetic Engineering, Collaborative Innovation Center for Genetics and Development, School of Life Sciences and Human Phenome Institute, Fudan University, China; State Key Laboratory of Genetic Engineering, Collaborative Innovation Center for Genetics and Development, School of Life Sciences and Human Phenome Institute, Fudan University, China; Ministry of Education Key Laboratory of Contemporary Anthropology, Department of Anthropology and Human Genetics, School of Life Sciences, Fudan University, China; Ministry of Education Key Laboratory of Contemporary Anthropology, Department of Anthropology and Human Genetics, School of Life Sciences, Fudan University, China; Ministry of Education Key Laboratory of Contemporary Anthropology, Department of Anthropology and Human Genetics, School of Life Sciences, Fudan University, China; Ministry of Education Key Laboratory of Contemporary Anthropology, Department of Anthropology and Human Genetics, School of Life Sciences, Fudan University, China; Ministry of Education Key Laboratory of Contemporary Anthropology, Department of Anthropology and Human Genetics, School of Life Sciences, Fudan University, China; Ministry of Education Key Laboratory of Contemporary Anthropology, Department of Anthropology and Human Genetics, School of Life Sciences, Fudan University, China; State Key Laboratory of Genetic Engineering, Collaborative Innovation Center for Genetics and Development, School of Life Sciences and Human Phenome Institute, Fudan University, China; State Key Laboratory of Genetic Engineering, Collaborative Innovation Center for Genetics and Development, School of Life Sciences and Human Phenome Institute, Fudan University, China; State Key Laboratory of Genetic Engineering, Collaborative Innovation Center for Genetics and Development, School of Life Sciences and Human Phenome Institute, Fudan University, China; State Key Laboratory of Genetic Engineering, Collaborative Innovation Center for Genetics and Development, School of Life Sciences and Human Phenome Institute, Fudan University, China; Ministry of Education Key Laboratory of Contemporary Anthropology, Department of Anthropology and Human Genetics, School of Life Sciences, Fudan University, China; State Key Laboratory of Genetic Engineering, Collaborative Innovation Center for Genetics and Development, School of Life Sciences and Human Phenome Institute, Fudan University, China; Institute for Six-sector Economy, Fudan University, China; Ministry of Education Key Laboratory of Contemporary Anthropology, Department of Anthropology and Human Genetics, School of Life Sciences, Fudan University, China; Ministry of Education Key Laboratory of Contemporary Anthropology, Department of Anthropology and Human Genetics, School of Life Sciences, Fudan University, China; Ministry of Education Key Laboratory of Contemporary Anthropology, Department of Anthropology and Human Genetics, School of Life Sciences, Fudan University, China; Shanghai Pudong Hospital, Fudan University Pudong Medical Center, China; State Key Laboratory of Genetic Engineering, Collaborative Innovation Center for Genetics and Development, School of Life Sciences and Human Phenome Institute, Fudan University, China; Fudan-Taizhou Institute of Health Sciences, China; State Key Laboratory of Genetic Engineering, Collaborative Innovation Center for Genetics and Development, School of Life Sciences and Human Phenome Institute, Fudan University, China; Key Laboratory of High-Altitude Environment and Genes Related to Diseases of Tibet Autonomous Region, School of Medicine, Xizang Minzu University, China; Key Laboratory of High-Altitude Environment and Genes Related to Diseases of Tibet Autonomous Region, School of Medicine, Xizang Minzu University, China; Institute of Cardiovascular Disease, Shandong Provincial Western Hospital, China; Institute of Cardiovascular Disease, Shandong Provincial Western Hospital, China; State Key Laboratory of Genetic Engineering, Collaborative Innovation Center for Genetics and Development, School of Life Sciences and Human Phenome Institute, Fudan University, China; Institute for Six-sector Economy, Fudan University, China; Research Unit of Dissecting the Population Genetics and Developing New Technologies for Treatment and Prevention of Skin Phenotypes and Dermatological Diseases (2019RU058), Chinese Academy of Medical Sciences, China; State Key Laboratory of Genetic Engineering, Collaborative Innovation Center for Genetics and Development, School of Life Sciences and Human Phenome Institute, Fudan University, China; Institute for Six-sector Economy, Fudan University, China; Research Unit of Dissecting the Population Genetics and Developing New Technologies for Treatment and Prevention of Skin Phenotypes and Dermatological Diseases (2019RU058), Chinese Academy of Medical Sciences, China; International Human Phenome Institutes, China

Environmental stresses, such as temperature, disease and altitude could induce systematic changes of biological systems which manifests as concerted responses across multiple systems within a certain period of time [1]. High altitude acclimatization (HAA) refers to a series of adaptive physiological responses to hypoxic stress. During these processes, several physiological systems are interwoven [2], such as respiratory and cardiovascular systems. In particular, to ensure high efficiency of blood gas exchange under hypoxia, hypoxic ventilation response increases the amount of inhaled oxygen, meanwhile pulmonary vasoconstriction enhances blood flow perfusion [3]. Therefore, HAA is a typical example to explore the concerted responses of multiple physiological systems. In practice, the concerted responses during HAA can be quantified by the correlation analysis of biological phenotypes.

To explore concerted responses to high altitude exposure, we herein applied composite phenotype analysis (CPA) on a longitudinal HAA study (Supplementary Fig. S1). Application of CPA on four-phase data (plain: Baseline; acute exposure: Acute; chronic exposure: Chronic; back to plain: De-acclimatization) were designed to capture dynamic responses of physiological systems and their interactions [4,5]. Single phenotype analysis indicated significant fluctuations of multisystem functions during HAA. To integrate the temporal dynamics of phenotypes, we integrated the four-dimensional phenotype across four phases as longitudinal phenotype and dissected their correlation structures. Composite phenotypes were extracted based on clusters of longitudinal phenotypes. Finally, the concerted responses of physiological systems were revealed by correlation network of composite phenotypes.

A total of 33 physiological phenotypes from 822 participants (subjects with cancer, diabetes, and coronary heart disease were not included in this study) were collected at four phases, including Lake Louise Score (LLS) questionnaire, biochemical, hematological and physical aspects (Supplementary Fig. S1, Supplementary Methods). The summary statistics showed that most phenotypes changed dramatically across four phases (Supplementary Table S1). For LLS questionnaire aspects, the high prevalence of acute mountain sickness (AMS) has been observed (47.45%) at Acute phase. For biochemical aspects, continuous increased aspartate aminotransferase (AST) was found, suggesting potential liver injury. At Chronic phase, both increased blood urea nitrogen (BUN) and serum creatinine (CREA) suggested the progressive decrease in glomerular filtration rate (GFR) and potential kidney function damage. For hematological aspects, increased red blood cell count (RBC), platelet count (PLT) and white blood cell count (WBC) showed that the hematopoietic system could be activated. For physical aspects, increased heart rate (HR), diastolic blood pressure (DBP) and systolic blood pressure (SBP) reflected that the heart was overloaded with increasing cardiac output. Thus, multiple physiological systems were dynamically changed during the process, and these systems should be taken into account for altitude acclimatization. However, it was unclear how the physiological systems interacted with each other during altitude acclimatization, and it was essential to dissect the relationships among these physiological systems.

Given the plasticity of phenotypes, the correlations of phenotypes were retained across four phases (Supplementary Fig. S2) resulting from the robustness of physiological systems. To incorporate the temporal dynamics, the same phenotype of the four phases was integrated as a longitudinal phenotype. Canonical correlation analysis (CCA) of longitudinal phenotype was performed to construct phenotype correlation network (Fig. 1A). To identify the longitudinal single phenotype structure, we then applied a fast-greedy clustering approach on 33 longitudinal single phenotypes at four phases, and finally obtained nine clusters (Fig. 1B).

**Figure 1.**
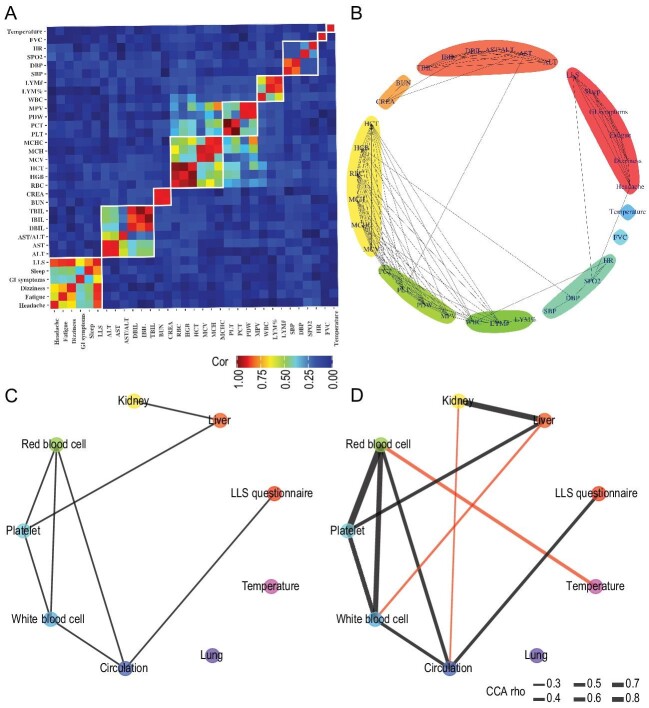
The correlation heatmap (A) and correlation network (B) of longitudinal phenotypes. The correlation heatmap of longitudinal single phenotypes (A) was filled with absolute value of CCA correlation coefficients. The clusters were identified by fast-greedy clustering method based on network (B). Networks of physiological systems induced by longitudinal single phenotypes (C) and composite phenotypes (D). The correlations were calculated by canonical correlation analysis and the associations between the longitudinal single phenotypes or composite phenotypes were shown based on the significance of their correlations. Compared with network of single phenotypes, the novel connections in the composite phenotype network are marked with red lines.

The nine composite phenotypes were defined as the set/combination of clustered single phenotypes (Table 1), which were highly consistent with the prior knowledge of the physiological classification. Therefore, these composite phenotypes were accordingly named by physiological knowledge labels (Table 1). Especially, for physical aspects, oxygen saturation (SPO_2_), heart rate (HR) and blood pressure (SBP, DBP) formed composite phenotype: Circulation. At Baseline phase, there are no strong correlations between SPO_2_, HR and blood pressure (SBP, DBP) under normoxia (Supplementary Fig. S3A and Supplementary Fig. S3B). However, at Acute phase, SPO_2_ was correlated with HR, and both HR and SPO_2_ were correlated with blood pressure (Fig. 1A and B). Therefore, it is reasonable to take these circulation related phenotypes as one composite phenotype.

**Table 1.** The detailed content of 9 composite phenotypes.

Composite phenotypes	Single phenotypes
LLS questionnaire	Headache, gastrointestinal symptoms, fatigue, dizziness, sleep, LLS
Liver	ALT, AST, AST/ALT, DBIL, IBIL, TBIL
Kidney	BUN, CREA
Red blood cell	HCT, HGB, RBC, MCH, MCV, MCHC
Platelet	PLT, PCT, PDW, MPV
White blood cell	WBC, LYM#, LYM%
Circulation	SPO_2_, HR, SBP, DBP
Lung	FVC
Temperature	Temperature

To investigate concerted responses among these composite phonotypes, we further adopted CCA to evaluate the correlations between composite phenotypes (Supplementary Table S1 and Fig. 1C and D). Compared with other dimensionality reduction approaches (PCA, etc.), CPA depicted more comprehensive and informative relationships between physiological systems (Supplementary Fig. S4 and Supplementary Methods). Comparing with the network of longitudinal single phenotypes, we found three new associations including the ones between Red Blood Cell and Temperature (rho = 0.365, *P*-value = 5.26E-09), between White Blood Cell and Liver (rho = 0.287, *P*-value = 2.43E-05), and between Kidney and Circulation (rho = 0.277, *P*-value = 3.67E-04) in the network of composite phenotypes. To further explore these three correlations, we constructed composite phenotype networks at each phase separately (Supplementary Fig. S5). The correlation between Kidney and Circulation was replicated at both Baseline and Acute phases, and the correlation between Red Blood Cell and Temperature was also found at Acute phase. However, without CPA, the correlation between White Blood Cell and Liver could not be observed at any phase (Supplementary Fig. S5).

Among all human physiological systems, the kidney played a crucial role in regulating body fluids, electrolyte and acid-base homeostasis after acute hypoxia exposure. And Sherpas demonstrated a larger plasma volume than Andeans, resulting in a comparable total blood volume at a lower hemoglobin concentration [6]. Thus, the Kidney and Circulation composite phenotype may be correlated through volume regulation in altitude acclimatization. It was also reported that hemoglobin dynamics [7] in Red Blood Cell were correlated with Temperature. And higher levels of White Blood Cell counts are associated with Liver diseases and enzymes [8]. Based on the above, these three novel associations could give us new insights to understand the potential mechanism of biological processes of altitude acclimatization.

Altitude acclimatization is the physiological process which takes place in the body on exposure to hypoxia at altitude, the most important change is the increase in breathing, and another is the well-known increase in hemoglobin concentration in the blood. As for altitude adaption, Tibetans have higher ventilation, higher oxygen saturation, lower pulmonary artery pressure and lower hemoglobin concentration compared to Han Chinese [9].

Although we collected multiple phenotypes covering the main physiological systems, considering the complexity of specific physiological systems, such as lung, the number of measured phenotypes were still limited. The phenotypes of individuals in this study were collected in the field and the total sample size was not very large, which may limit extrapolation of our results. To achieve more comprehensive analyses of altitude acclimatization, more frequent data collection would provide more detailed information instead of just four time-points. Molecular phenotypes like epigenome, transcriptome, proteome, metabolome, metagenome and other omics could also provide more valuable information of different physiological systems of altitude acclimatization.

In summary, we applied CPA to reveal potential concerted responses of physiological systems to high altitude exposure. CPA can be considered as a general approach of system biology and phenomics research, especially in large-scale longitudinal cohort studies [10].

## Supplementary Material

nwad053_Supplemental_Files

## References

[1] Killen SS , Marras S, Metcalfe NB et al. Trends Ecol Evol 2013; 28: 651–8. 10.1016/j.tree.2013.05.005 23756106

[2] West J , Schoene R, Luks A et al. High Altitude Medicine and Physiology 5E. London: CRC Press; 2012.

[3] Luks AM , Hackett PH. N Engl J Med 2022; 386: 364–73. 10.1056/NEJMra2104829 35081281

[4] Li Y , Ma Y, Wang K et al. Phenomics 2021; 1: 3–14. 10.1007/s43657-020-00005-8 36939745 PMC9584130

[5] Wang K , Zhang M, Li Y et al. J Headache Pain 2018; 19: 59. 10.1186/s10194-018-0878-7 PMC6060196 30046908

[6] Stembridge M , Williams AM, Gasho C et al. Proc Natl Acad Sci USA 2019; 116: 16177–9. 10.1073/pnas.1909002116 31358634 PMC6697886

[7] Stadler AM , Digel I, Artmann GM et al. Biophys J 2008; 95: 5449–61. 10.1529/biophysj.108.138040 18708462 PMC2586580

[8] Lee YJ , Lee HR, Shim JY et al. Dig Liver Dis 2010; 42: 888–94. 10.1016/j.dld.2010.04.005 20472517

[9] Xu S , Li S, Yang Y et al. Mol Biol Evol 2011; 28: 1003–11. 10.1093/molbev/msq277 20961960

[10] Hao M , Zhang H, Hu Z et al. Aging Cell 2021; 20: e13519. 10.1111/acel.13519 34825761 PMC8672793

