# Supplementary material for: The composite phenotype analysis identifies potential concerted responses of physiological systems to high altitude exposure
Source: Natl Sci Rev. 2023 Mar 1;10(5):nwad053. doi: 10.1093/nsr/nwad053 (PMC10089582; doi:10.1093/nsr/nwad053)
Supplement: nwad053_Supplemental_Files [file nwad053_supplemental_files.zip › Supplementary-Fig_S1.pdf]

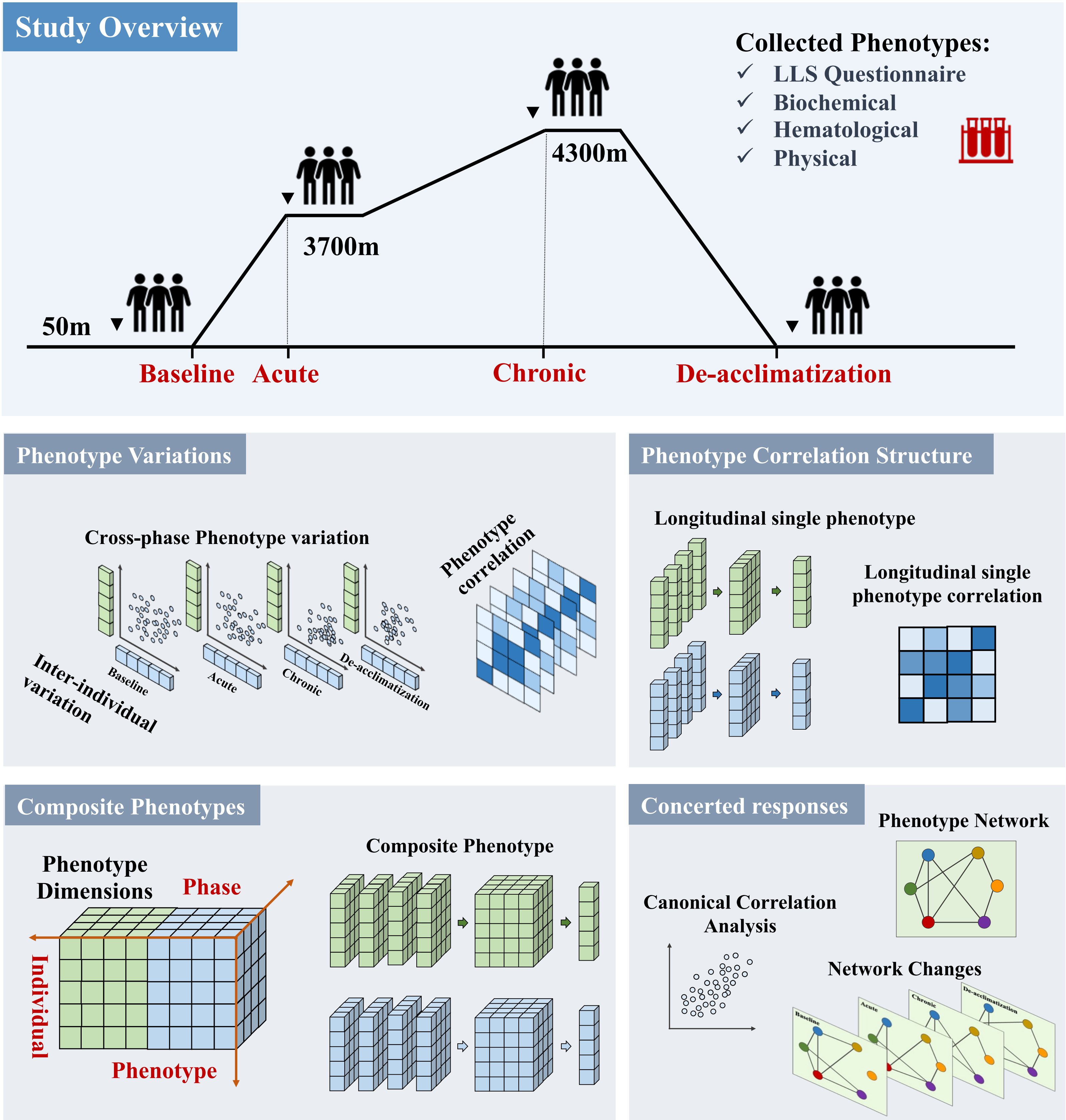
Study Overview
Collected Phenotypes:
✓ LLS Questionnaire
✓ Biochemical
✓ Hematological
✓ Physical
4300m
3700m
50m
Baseline
Acute
Chronic
De-acclimatization
Phenotype Variations
Cross-phase Phenotype variation
Phenotype correlation
Baseline
Acute
Chronic
De-acclimatization
Inter-individual variation
Phenotype Correlation Structure
Longitudinal single phenotype
Longitudinal single phenotype correlation
Composite Phenotypes
Phenotype Dimensions
Phase
Individual
Phenotype
Composite Phenotype
Concerted responses
Phenotype Network
Canonical Correlation Analysis
Network Changes
Baseline
Acute
Chronic
De-acclimatization
