# Supplementary material for: The composite phenotype analysis identifies potential concerted responses of physiological systems to high altitude exposure
Source: Natl Sci Rev. 2023 Mar 1;10(5):nwad053. doi: 10.1093/nsr/nwad053 (PMC10089582; doi:10.1093/nsr/nwad053)
Supplement: nwad053_Supplemental_Files [file nwad053_supplemental_files.zip › Supplementary-Fig_S6.pdf]

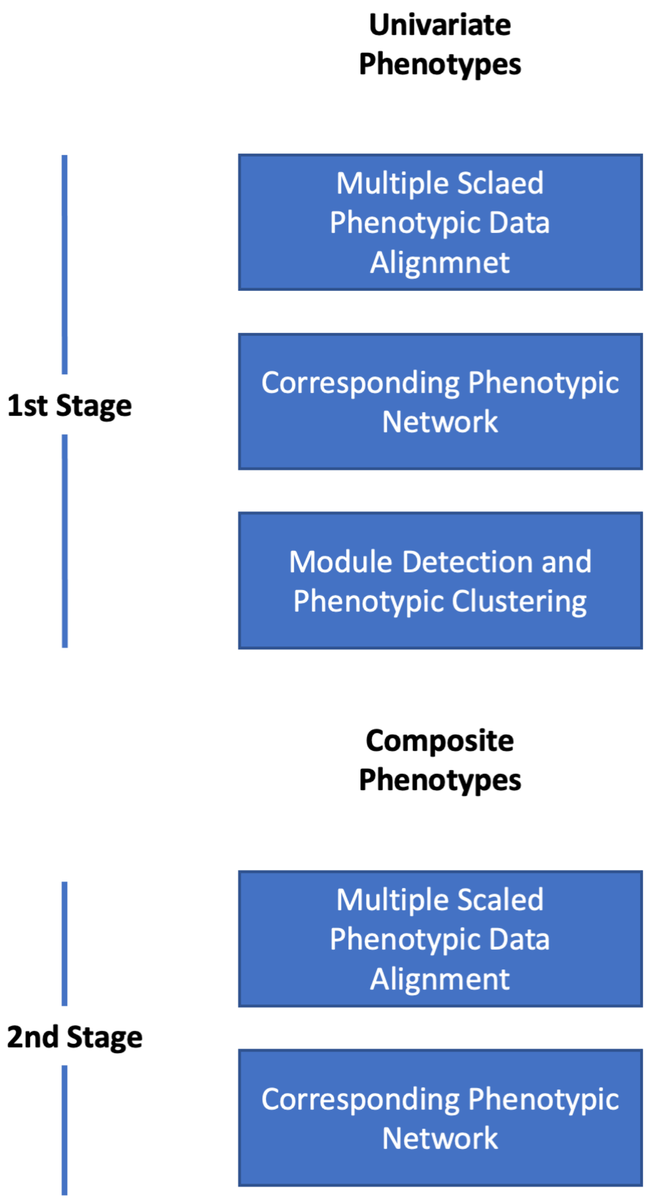
Univariate Phenotypes
1st Stage
Multiple Sclaed Phenotypic Data Alignmnet
Corresponding Phenotypic Network
Module Detection and Phenotypic Clustering
Composite Phenotypes
2nd Stage
Multiple Scaled Phenotypic Data Alignment
Corresponding Phenotypic Network
