# Supplementary material for: The composite phenotype analysis identifies potential concerted responses of physiological systems to high altitude exposure
Source: Natl Sci Rev. 2023 Mar 1;10(5):nwad053. doi: 10.1093/nsr/nwad053 (PMC10089582; doi:10.1093/nsr/nwad053)
Supplement: nwad053_Supplemental_Files [file nwad053_supplemental_files.zip › Supplementary-Methods.2023.2.26.docx]

**Methods**

**Phenotype collection and measurement**

In this cohort, a total of 822 male healthy Chinese Han volunteers aged from 17 to 36 (21.93 ± 3.17) years old were recruited, the subjects with cancer, diabetes, and coronary heart disease were not included in this study. The researches were approved by the Ethics Committee of School of Life Science, Fudan University and written informed consent was obtained from each participant and their guardians over 18 years old. We collected demographic, physiological, blood and LLS phenotypes at 4 time points (**Fig. S1**) referring to Baseline (plain, 50 meters), Acute phase (1-3 days after arrival at 3700 meters high by train), Chronic phase (a month after arrival at 4300 meters high by train) and De-acclimatization (a month after back to plain by train). We collected the physiological, biochemical, blood phenotypes and LLS questionnaires at the four periods. The physiological phenotypes included heart rate (HR, beats/min), diastolic blood pressure (DBP, mmHg), systolic blood pressure (SBP, mmHg), forced vital capacity (FVC, ml) and oxygen saturation (SPO_2_, %). The biochemical phenotypes included Lalanine aminotransferase (ALT, U/L), aspartate aminotransferase (AST, U/L), blood urea nitrogen (BUN, mmol/L), serum creatinine (CREA, umol/L), total serum bilirubin (TBIL, umol/L), direct serum bilirubin (DBIL, umol/L) and indirect serum bilirubin (IBIL, umol/L). The blood phenotypes included red blood cell count (RBC, × 10^12^/L), hemoglobin (HGB, g/L), hematocrit (HCT, %), mean corpuscular volume (MCV, fL), mean corpuscular hemoglobin (MCH, pg), mean corpuscular hemoglobin concentration (MCHC, g/L), white blood cell count (WBC, × 10^9^/L), lymphocyte percentage (LYM%), absolute lymphocyte count (LYM#), platelet count (PLT, × 10^9^/L), plateletcrit (PCT, fL), mean platelet volume (MPV, fL), platelet distribution width (PDW, fL). The LLS questionnaire consisted of five items: headache, dizziness, gastrointestional symptoms, fatigue/weekness and difficulty sleeping.

All the subjects (822 samples, 33 phenotypes) were measured by physicians in Shandong Provincial Western Hospital, who were previously trained to administer both the questionnaire and the physical examination (1, 2). Systolic blood pressure (SBP) and diastolic blood pressure (DBP) were calculated by mean of two rounds of measurement of a standardized mercury sphygmomanometer. Maximal vital capacity (FVC) was measured by SPIDA5. Heart rate (HR) was determined by measuring radial pulse twice, and SPO_2_ was measured by Nellcor NPB-40. The body temperature (Temperature) was measured by a thermometer. The blood specimens were drawn after overnight fasting for complete blood count measurement by a three-classification haemacytometer analyzer (Model CA-800; CIS, Japan). Blood biochemical indices were measured using an automatic biochemical analyzer (Model 7060; Hitachi Ltd., Japan). The LLS questionnaire consists of five items: headache, dizziness, gastrointestinal symptoms, fatigue/weakness, and difficulty sleeping. Each item was rated on a four-point scale (0 = not at all, 1 = mild, 2 = moderate, and 3 = severe). Single item scores are added up, and the maximal score is 15.

**Composite phenotype analysis**

We have developed an analytical framework, called Composite Phenotype Analysis, that strikes a balance within and cross biosystems. With correlation network techniques, we build a network describing the interaction between phenotypes. The network can be used for finding submodules of highly interacted phenotypes which indicates the phenotypes within the same biosystems and quantify these modules as the measurement of composite phenotype. The concerted response in different scale could be identified by further summarizing the interaction cross phenotypes or composite phenotypes as well as the interaction between composite phenotype and phenotype. The biological utility of the Composite Phenotype Analysis is demonstrated by a longitudinal data study of 822 Han Chinese young males each with 33 physiological phenotypes of altitude acclimatization including four phases as Baseline, Acute, Chronic and De-acclimatization. The output of the analysis is a three scales network describing that how biosystem and phenotypes are concerted during altitude acclimatization.

Two stage-composite phenotype analysis was used to detect and analyze the concerted response between phenotypes. It consists of three components (**Fig. S6**). Data alignment helps merge the phenotypes in multiple scale. Then the corresponding network provides the interactions between phenotypes. Module detection points out the construction of composite phenotypes. After that, the same process will be executed again for revealing potential concerted responses between composite phenotypes.

**Matrix Factorization and Canonical Correlation Analysis**

There is often a strong correlation between phenotypes in different periods. In order to eliminate the impact of these autocorrelations on the analysis, we will first regularize the data through matrix factorization.

Consider matrix $D_{N\times T}$ represents specific phenotype information from N samples and is measured cross T periods. The n^th^ row of the matrix is a vector of T measurements of the specific phenotype for the n^th^ sample and the t^th^ column of the matrix is a vector of N measurements of the specific phenotype at the t^th^ moment cross all the samples. To perform a matrix factorization, we transform the matrix A into the production of two lower rank matrices.

Assume the rank of matrix $D_{N\times T}$ is $R$. $R\leq min(N,T)$. Consider matrices $W_{N\times R}$ and $H_{R\times T}$, and the matrix factorization attempts to minimize the approximation error:

$$\min_{W,H} {||D-WH||}^{2}$$

By solving the optimization problem, we will have the rank-R matrix W as a multidimensional vector representing the phenotype data in different scale.

Then we construct the phenotypic corresponding network with pairwise canonical correlation analysis for different regularized representers of the phenotypes. Consider $X_{N\times T_{1}}$ is a phenotype matrix of N samples cross T_1_ periods and $Y_{N\times T_{2}}$ is another phenotype matrix of N samples cross T_2_ periods. Let $Z_{N\times\left( T_{1}+T_{2} \right)}={[X^{T},Y^{T}]}^{T}$ is the joint matrix of the two metrics. Then we can have the joint covariance matrix as

$$\Sigma_{ZZ}=\left[ \begin{matrix} \Sigma_{XX} & \Sigma_{YX} \\ \Sigma_{XY} & \Sigma_{YY} \end{matrix} \right]$$

Let

$$R^{2}={\Sigma_{YY}}^{-1/2}\Sigma_{YX}{\Sigma_{XX}}^{-1/2}{\Sigma_{XX}}^{-1/2}\Sigma_{XY}{\Sigma_{YY}}^{-1/2}$$

And

$$K={\Sigma_{XX}}^{-1/2}\Sigma_{XY}{\Sigma_{YY}}^{-1/2}=UDV^{T}$$

Where $D_{R}=diag(d_{1},\ldots,d_{q})$ and $q=min(T_{1},T_{2})$.

The canonical vectors are:

$$A= \Sigma_{XX}U$$

$${B= \Sigma}_{YY}V$$

The canonical correlation vector is:

$$CC= {[d}_{1},\ldots,d_{q}]$$

We can have its summation of square value to quantify the correlation coefficient between the two multivariate variables.

To test the association between the two representers. The null hypothesis of no association can be formulated as

$$H0: \Sigma_{XY}=0$$

And the likelihood ratio test statistic for testing the association, as Bartlett(1939) suggested, is:

$$T_{CCA}=-[N-(q+3)/2]\sum log(1-{CC}^{2})$$

**Module detection with the fast-greedy algorithm**

Module Detection with spectral clustering followed by the fast greedy algorithm would be applied to cluster the phenotypes into several submodules. Phenotypes in the same group are more similar to each other in either numerical or fluctuant patterns than to the phenotypes from the other groups.

Greedy algorithm is an algorithm that follows the problem-solving heuristic of making the locally optimal choice at each stage^29^ with the intent of finding a global optimum. The algorithm has very prominent effects on the optimization of submodule detection. Traditionally, the fast-greedy algorithm is applied to the adjacent matrix $A_{X\times Y}$ of the network^30^, where

$$A_{xy}=\left\{ \begin{aligned} 1 \\ 0 \end{aligned} {phenotype x and y is connected in the network \atop otherwise} \right.$$

While with the contribution of CCA correlation matrix, we could redefine the matrix as:

$$A_{xy}=\left\{ \begin{aligned} {CCA}_{x,y} \\ 0 \end{aligned} {phenotype x and y is canonical correlated significantly \atop otherwise} \right.$$

And in this way, compared with the traditional submodule detection with fast greedy algorithm, we propose a weighted submodule detection method for the problem.

Consider the correlation matrix of CCA coefficient cross multi phenotypes $A_{X\times Y}$. $A_{xy}$is the correlation coefficient between phenotype x and phenotype y. $C_{x}$ and $C_{y}$ is the submodule which the phenotype x and phenotype y belong to. We define the measurement of the modularity as:

$$S=\frac{1}{2m}\sum_{x,y} [A_{xy}-\frac{k_{x}k_{y}}{2m}]\delta{(C}_{x},C_{y})$$

Where m is the number of edges in $A_{xy}$, calculated as ${m=\frac{1}{2}\Sigma}_{x,y}A_{xy}$; $k_{x}{=\Sigma}_{y}A_{xy}$ and $k_{y}{=\Sigma}_{x}A_{xy}$ is the degree of phenotype x and y, $\frac{k_{x}k_{y}}{2m}$ is the probability of the existence of an edge between $C_{x}$ and $C_{x}$ at random.

Thus, we turn the clustering problem into an optimization problem and obtain the best classification method by finding the largest S.

**Stability Test**

We randomly selected 200, 400 and 600 samples for the analysis and repeat our process for 1000 experiments. The frequency of significant connection between specific phenotypes is calculated as the evaluation of the stability of our corresponding network (**Table S2, Table S3**) .

***Advantages of longitudinal single phenotypes***

To explore the advantages of longitudinal single phenotypes, we also investigate the clustering results of original single phenotypes at four phases (**Fig. S3A, 3B**). We found that hypoxic environmental stress could reveal, mask and modulate the relationships among the single phenotypes. For example, the relationships between Red Blood Cell related phenotypes (HCT, RBC, HGB, MCH, MCV, MCHC) and Platelet related phenotypes (PCT, PLT, PDW, MPV) were revealed throughout altitude acclimatization. At Baseline phase, they were all grouped into one cluster, and separated into different clusters in other phases. BUN was strongly correlated with CREA at Baseline while the relationship between BUN and CREA was masked at Acute phase (**Fig. S3C**). And they were always clustered together in Chronic and De-acclimatization phases. The relationship between HR and SPO_2_ was modulated by hypoxia at Acute phase (**Fig. S3D**), while HR was independent of SPO_2_ at Baseline phase. Furthermore, we compared the structures by aligning the clustering results of each phase and changes to the integrated results of four phases (**Fig. S3B**). To quantify the similarities among clustering results, the normalized mutual information (NMI) was applied (3) and it showed high concordance among the results (**mean NMI = 0.874**). The high NMI value indicated that the clustering results of longitudinal single phenotypes considering the temporal dynamics at each phase was consistent with each other. Thus, it’s reasonable to integrate the same single phenotype as a longitudinal single phenotype.

***Benchmark of composite phenotype analysis***

To comprehensively evaluate the results of composite phenotypes, we included four dimension reduction methods, including principal components analysis (PCA) (4), Locally Linear Embedding (LLE) (5), Nonnegative Matrix Factorization (NMF) (6) and Multiple Dimensional Scaling (MDS) (7). Compared with CPA, these methods are unsupervised and some information of phenotypes are inevitably lost (**Fig. S4**). There were 2, 1, 3, 3 significant associations using PCA, LLE, NMF and MDS respectively. Some associations were also revealed, however the p values failed to pass multiple testing correction. Therefore, we employed the phenotype network of single phenotype and composite phenotype to explore the changes of relationships between physiological systems in high altitude acclimatization.
 We also compared the correlation networks depicted by composite phenotypes with that of single phenotypes at four phases respectively (**Fig. S5**). At Baseline phase (**Fig. S5A, 5E**), the associations between **Kidney** and **Circulation** (rho = 0.166, p-value = 4.36E-05) and between **Platelet** and **Circulation** (rho = 0.193, p-value = 2.8E-04) were identified by composite phenotypes. Similarly, many more associations were identified by composite phenotypes at other three phases, including the one between **Kidney** and **LLS questionnaire** (rho = 0.181, p-value = 6.99E-04) at Acute phase (**Fig. S5B, 5F**), the one between **White Blood Cell** and **Circulation** (rho = 0.191, p-value = 6.01E-04) at Chronic phase (**Fig. S5C, 5G**), and the ones between **White Blood Cell** and **Circulation** (rho = 0.291, p-value = 9.77E-15) and between **LLS Questionnaire** and **Circulation** (rho = 0.22, p-value = 1.09E-04) at De-acclimatization phase (**Fig. S5D, 5H**). Therefore, compared with single phenotypes, composite phenotypes could identify additional associations between these physiological systems. At the same time, some associations could be observed in single phenotypic network but perform not as significant as those with composite phenotype analysis due to the degree of freedom (8), which indicates the instability of these links generally.

The associations of composite phenotypes changed significantly due to the change of plateau environment (**Fig. S5E-5H**). The increased or reduced associations between the composite phenotypes under new environment resulted from adaptive adjustments of physiological systems. In the process from Baseline to Acute (**Fig. S5E, 5F**), the associations between **Red Blood Cell** and **Circulation**, between **Platelet** and **Circulation,** and between **Kidney** and **Liver** were masked. Instead, the associations between **LLS Questionnaire** and **Kidney** (rho = 0.181, p-value = 6.99E-04), between **LLS Questionnaire** and **Circulation** (rho = 0.298, p-value = 8.8E-10), and between **Red Blood Cell** and **Temperature** (rho = 0.292, p-value = 8.26E-14) were revealed. In the process from Acute to Chronic (**Fig. S5F, 5G**), the above added associations from Baseline to Acute were masked, and associations between **Platelet** and **White Blood Cell**, and between **Kidney** and **Circulation** were also masked. The associations between **Red Blood Cell** and **Liver** (rho = 0.239, p-value = 5.73E-04), between **Platelet** and **Liver** (rho = 0.265, p-value = 1.46E-08), between **Kidney** and **Liver** (rho = 0.268, p-value = 6.95E-10), and between **White Blood Cell** and **Circulation** (rho = 0.191, p = 6.01E-04) were revealed. In the process from Chronic to De-acclimatization (**Fig. S5G, 5H**), the **Liver** related associations were masked, while the association between **Circulation** and **LLS Questionnaire** (rho = 0.22, p-value = 1.09E-04) was revealed. In summary, the additional associations of composite phenotypes suggested the concerted responses between physiological systems reacting to environmental stress. The dynamic correlation networks of composite phenotypes provided a reference of physiological systems throughout high altitude acclimatization.

In summary, the correlation networks of physiological systems depicted by composite phenotypes could detect more associations compared with the networks of single phenotypes under a specific environmental condition. It shows the advantages of our method to find more hidden interactions. At the same time, the differences in phenotypic networks in different phases also point out the necessity to integrate the phenotypes longitudinally so as to denoise the impact of specific environmental disturbances for in-depth analysis.

Composite phenotypes can be identified by some clustering algorithms or prior knowledge. In practice, the data-driven clustering algorithms are more objective and reliable than prior knowledge classification. Multivariate statistical methods had been applied to extract composite phenotypes, such as Principle Component Analysis (PCA) (9), Canonical Correlation Analysis (CCA) (10) and Partial Least-Squares (PLS) (1). Both PCA and PLS take fixed latent variable scores as the composite phenotypes for each phenotype cluster, but they are less informative and explainable due to reduced data and fixed internal relationship. Compared with four dimension reduction methods (PCA, LLE, NMF and MDS), CPA revealed more informative associations between physiological systems (**Fig. S4**). And the composite phenotype referred as a set of related single phenotypes contained all linear formulas of the single phenotype with CCA as an interface.

**Reference**

1. Li, Y, Ma, Y, Wang, K*, et al.* Using Composite Phenotypes to Reveal Hidden Physiological Heterogeneity in High-Altitude Acclimatization in a Chinese Han Longitudinal Cohort. *Phenomics*. 2021.

2. Wang, K, Zhang, M, Li, Y*, et al.* Physiological, hematological and biochemical factors associated with high-altitude headache in young Chinese males following acute exposure at 3700 m. *The journal of headache and pain*. 2018; **19**(1): 59.

3. Knops, ZF, Maintz, JB, Viergever, MA*, et al.* Normalized mutual information based registration using k-means clustering and shading correction. *Med Image Anal*. 2006; **10**(3): 432-9.

4. Yi, H, Wo, H, Zhao, Y*, et al.* Comparison of dimension reduction-based logistic regression models for case-control genome-wide association study: principal components analysis vs. partial least squares. *J Biomed Res*. 2015; **29**(4): 298-307.

5. Liu, X, Tosun, D, Weiner, MW*, et al.* Locally linear embedding (LLE) for MRI based Alzheimer's disease classification. *Neuroimage*. 2013; **83**: 148-57.

6. Zetlaoui, M, Feinberg, M, Verger, P*, et al.* Extraction of food consumption systems by nonnegative matrix factorization (NMF) for the assessment of food choices. *Biometrics*. 2011; **67**(4): 1647-58.

7. Aflalo, Y, Dubrovina, A, Kimmel, R. Spectral generalized multi-dimensional scaling. *International Journal of Computer Vision*. 2016; **118**(3): 380-92.

8. Walker, HM. Degrees of freedom. *Journal of Educational Psychology*. 1940; **31**(4): 253.

9. Ried, JS, Jeff, MJ, Chu, AY*, et al.* A principal component meta-analysis on multiple anthropometric traits identifies novel loci for body shape. *Nat Commun*. 2016; **7**: 13357.

10. Hu, Z, Jiao, R, Wang, P*, et al.* Shared Causal Paths underlying Alzheimer's dementia and Type 2 Diabetes. *Sci Rep*. 2020; **10**(1): 4107.

**Supplementary Figure Legends:**

**Fig. S1.** The design of the longitudinal high altitude acclimatization cohort (top) and workflow of composite phenotype analysis.

**Fig. S2.** The correlation heatmaps of single phenotypes at four phases (A: Baseline; B: Acute; C: Chronic; D: De-acclimatization). The heatmaps were filled with absolute value of Spearman correlation coefficients. The correlation between the correlation matrices were calculated using Mantel test.

**Fig. S3.** The structure of original single phenotypes at four phases. And different clusters were represented by different color (A). The alignment of single phenotypes and changes clustering results with the integrated longitudinal phenotype results of four phases (B). The remarkable cases of phenotype relationships regulated by hypoxia environment (C, D).

**Fig. S4.** The physiological system correlation networks depicted by PCA, LLE, NMF, MDS.

**Fig. S5.** The physiological system correlation networks depicted by single phenotypes (A-D) and composite phenotypes at four phases (E-H). A & E: Baseline; B & F: Acute; C & G: Chronic; D & H: De-acclimatization.

**Fig. S6.** The workflow of two stage-composite phenotype analysis.
