# Supplementary material for: The composite phenotype analysis identifies potential concerted responses of physiological systems to high altitude exposure
Source: Natl Sci Rev. 2023 Mar 1;10(5):nwad053. doi: 10.1093/nsr/nwad053 (PMC10089582; doi:10.1093/nsr/nwad053)
Supplement: nwad053_Supplemental_Files [file nwad053_supplemental_files.zip › Supplementary-Table_S1.docx]

**Table S1.** The comparison of 33 single phenotypes at 4 phases of altitude acclimatization.

| **Phenotype** | **Baseline** | |  | **Acute** | |  | **Chronic** | |  | **De-acclimatization** | |
| --- | --- | --- | --- | --- | --- | --- | --- | --- | --- | --- | --- |
| **Questionnaire** | **N** | **Ratio, %** |  | **N** | **Ratio, %** |  | **N** | **Ratio, %** |  | **N** | **Ratio, %** |
| Headache | 100 | 16.7 |  | 477^*^ | 61.2 |  | 155^*#^ | 20.8 |  | 48^*#&^ | 7.2 |
| Dizziness | 137 | 22.9 |  | 557^*^ | 71.6 |  | 290^*#^ | 39.0 |  | 216^*#&^ | 32.3 |
| Fatigue | 123 | 20.6 |  | 512^*^ | 66.0 |  | 225^*#^ | 30.2 |  | 107^*#&^ | 16.0 |
| Gastrointestinal Symptoms | 38 | 6.4 |  | 163^*^ | 21.2 |  | 122^*#^ | 16.4 |  | 45^*#&^ | 6.8 |
| Sleep | 69 | 11.6 |  | 323^*^ | 41.6 |  | 184^*#^ | 24.7 |  | 99^*#&^ | 14.8 |
|  | **Mean** | **Sd** |  | **Mean** | **Sd** |  | **Mean** | **Sd** |  | **Mean** | **Sd** |
| LLS | 5.84 | 1.56 |  | 7.99 | 2.15 |  | 6.41 | 1.74 |  | 5.84 | 1.38 |
| **Biochemical** |  |  |  |  |  |  |  |  |  |  |  |
| ALT, U/L | 18.79 | 7.75 |  | 9.95^*^ | 6.29 |  | 14.88^*#^ | 9.08 |  | 12.26^*#&^ | 12.57 |
| AST, U/L | 15.49 | 5.75 |  | 33.92^*^ | 14.74 |  | 46.24^*#^ | 15.25 |  | 24.21^*#&^ | 21.51 |
| AST/ALT | 0.85 | 0.19 |  | 4.31^*^ | 3.08 |  | 3.94^*^ | 2.42 |  | 2.45^*#&^ | 1.13 |
| TBIL, umol/L | 11.89 | 1.69 |  | 13.83^*^ | 5.57 |  | 13.72^*^ | 6.81 |  | 12.68^*#&^ | 5.96 |
| DBIL, umol/L | 2.68 | 0.58 |  | 5.04^*^ | 1.58 |  | 5.7^*#^ | 1.71 |  | 4.75^*#&^ | 1.61 |
| IBIL, umol/L | 9.22 | 1.20 |  | 8.8^*^ | 4.90 |  | 7.71^*#^ | 4.18 |  | 7.94^*#^ | 5.45 |
| BUN, mmol/L | 5.07 | 1.13 |  | 5.03 | 1.14 |  | 6.2^*#^ | 1.17 |  | 5.54^*#&^ | 1.20 |
| CREA, umol/L | 58.31 | 10.39 |  | 102.06^*^ | 13.70 |  | 113.04^*#^ | 12.24 |  | 118.34^*#&^ | 12.07 |
| **Hematological** |  |  |  |  |  |  |  |  |  |  |  |
| WBC, *10^9^ | 6.21 | 1.38 |  | 8.59^*^ | 2.33 |  | 8.2^*#^ | 1.70 |  | 6.58^*#&^ | 1.60 |
| LYM%, % | 36.07 | 7.64 |  | 35.67 | 8.82 |  | 40.68^*#^ | 10.77 |  | 33.66^*#&^ | 8.88 |
| LYM#, *10^9^ | 2.20 | 0.53 |  | 2.98^*^ | 0.82 |  | 3.31^*#^ | 0.99 |  | 2.16^#&^ | 0.61 |
| RBC, *10^2^ | 4.87 | 0.39 |  | 5.25^*^ | 0.70 |  | 5.73^*#^ | 0.48 |  | 4.81^#&^ | 0.69 |
| HGB, g/L | 150.18 | 10.15 |  | 162.94^*^ | 23.54 |  | 179.54^*#^ | 13.72 |  | 136.98^*#&^ | 20.12 |
| HCT, % | 0.44 | 0.03 |  | 0.45 | 0.06 |  | 0.5^*#^ | 0.04 |  | 0.43^*#&^ | 0.06 |
| MCV, fL | 90.74 | 4.98 |  | 86.11^*^ | 5.50 |  | 87.37^*#^ | 4.96 |  | 90.16^*#&^ | 5.22 |
| MCH, pg | 30.96 | 2.31 |  | 31.05 | 2.52 |  | 31.37^*#^ | 2.22 |  | 28.49^*#&^ | 1.85 |
| MCHC, g/L | 341.14 | 18.02 |  | 360.56^*^ | 18.06 |  | 358.93^*#^ | 16.45 |  | 315.84^*#&^ | 9.26 |
| PLT, *10^9^ | 207.55 | 42.33 |  | 248.54^*^ | 67.17 |  | 260.44^*#^ | 51.94 |  | 214.34^#&^ | 58.32 |
| PCT, % | 2.01 | 0.42 |  | 2.59^*^ | 0.73 |  | 2.73^*#^ | 0.52 |  | 2.21^*#&^ | 0.60 |
| MPV, fL | 9.75 | 1.23 |  | 10.47^*^ | 0.63 |  | 10.54^*#^ | 0.62 |  | 10.33^*#&^ | 0.61 |
| PDW, % | 13.73 | 2.20 |  | 17.18^*^ | 2.45 |  | 18.03^*#^ | 2.54 |  | 16.65^*#&^ | 2.48 |
| **Physical** |  |  |  |  |  |  |  |  |  |  |  |
| SBP, mmHg | 110.80 | 10.60 |  | 118.32^*^ | 12.06 |  | 124.19^*#^ | 12.81 |  | 114.36^*#&^ | 12.44 |
| DBP, mmHg | 73.14 | 8.77 |  | 81.13^*^ | 9.84 |  | 75.8^*#^ | 9.57 |  | 72.23^#&^ | 9.32 |
| HR, beats/min | 66.54 | 9.71 |  | 82.65^*^ | 11.97 |  | 87.16^*#^ | 11.02 |  | 68.6^*#&^ | 9.76 |
| SPO_2_, % | 97.73 | 2.22 |  | 88.44^*^ | 4.24 |  | 85.84^*#^ | 3.80 |  | 98.17^*#&^ | 1.12 |
| FVC, L | 444.07 | 38.95 |  | 427.84^*^ | 60.17 |  | 412.71^*#^ | 56.11 |  | 403.71^*#&^ | 55.41 |
| Temperature, ℃ | 36.22 | 0.12 |  | 36.81^*^ | 0.69 |  | 36.38^*#^ | 0.29 |  | 36.26^*#&^ | 0.16 |

Five traits of LLS Questionnaire were shown with number of occurrences and ratio. Wilcoxon Rank-Sum Test was used to compare the mean of the two groups. The significance was shown with three symbols (*: p<0.05 versus Baseline, #: p <0.05 versus Acute, &: p<0.05 versus Chronic)
