# Supplementary material for: The composite phenotype analysis identifies potential concerted responses of physiological systems to high altitude exposure
Source: Natl Sci Rev. 2023 Mar 1;10(5):nwad053. doi: 10.1093/nsr/nwad053 (PMC10089582; doi:10.1093/nsr/nwad053)
Supplement: nwad053_Supplemental_Files [file nwad053_supplemental_files.zip › Supplementary-Table_S2.docx]

**Table S2.** **Canonical correlation summary of phenotypic network in different scale.**

| **Composite Phenotypes** | **From** | **To** | **Max**  **CCA Rho** | **Average**  **CCA Rho** | **Stability** | **Pvalue** |
| --- | --- | --- | --- | --- | --- | --- |
| **Dynamic (4 time points)** | Kidney | Circulation | 0.30 | 0.17 | 63.4% | 6.00E-03 |
|  | Liver | Kidney | 0.71 | 0.29 | 99.1% | 3.60E-57 |
|  | LLS questionnaire | Circulation | 0.38 | 0.20 | 79.3% | 1.25E-04 |
|  | Platelet | Liver | 0.39 | 0.21 | 80.1% | 3.40E-07 |
|  | Red blood cell | Circulation | 0.41 | 0.21 | 82.4% | 2.63E-07 |
|  | Red blood cell | Platelet | 0.88 | 0.36 | 99.8% | 5.81E-271 |
|  | Temperature | Red blood cell | 0.29 | 0.22 | 54.7% | 2.58E-02 |
|  | White blood cell | Circulation | 0.38 | 0.18 | 77.6% | 2.39E-07 |
|  | White blood cell | Platelet | 0.39 | 0.18 | 80.5% | 2.17E-09 |
|  | White blood cell | Red blood cell | 0.50 | 0.26 | 91.5% | 1.24E-27 |
| **Stage 1 (Baseline)** | Kidney | Circulation | 0.16 | 0.13 | 55.7% | 1.71E-04 |
|  | Liver | Kidney | 0.70 | 0.39 | 98.9% | 8.80E-111 |
|  | Platelet | Circulation | 0.19 | 0.09 | 57.0% | 2.80E-04 |
|  | Red blood cell | Circulation | 0.27 | 0.14 | 64.4% | 3.65E-09 |
|  | Red blood cell | Platelet | 0.86 | 0.35 | 99.8% | 2.38E-245 |
|  | White blood cell | Platelet | 0.31 | 0.16 | 69.1% | 9.85E-15 |
|  | White blood cell | Red blood cell | 0.30 | 0.16 | 67.9% | 1.42E-11 |
| **Stage 2 (Acute)** | Kidney | Circulation | 0.19 | 0.12 | 57.1% | 9.05E-05 |
|  | LLS questionnaire | Circulation | 0.30 | 0.12 | 67.5% | 1.11E-09 |
|  | LLS questionnaire | Kidney | 0.18 | 0.13 | 56.6% | 7.79E-04 |
|  | Red blood cell | Platelet | 0.48 | 0.25 | 89.9% | 2.24E-50 |
|  | Temperature | Red blood cell | 0.15 | 0.15 | 54.8% | 6.90E-03 |
|  | White blood cell | Platelet | 0.26 | 0.13 | 62.8% | 1.74E-09 |
|  | White blood cell | Red blood cell | 0.38 | 0.24 | 78.5% | 1.05E-29 |
| **Stage 3 (Chronic)** | Liver | Kidney | 0.27 | 0.18 | 64.3% | 6.95E-10 |
|  | Platelet | Liver | 0.27 | 0.13 | 63.8% | 1.46E-08 |
|  | Red blood cell | Liver | 0.24 | 0.09 | 60.9% | 5.73E-04 |
|  | Red blood cell | Platelet | 0.52 | 0.28 | 93.2% | 1.03E-69 |
|  | White blood cell | Circulation | 0.19 | 0.09 | 57.1% | 6.01E-04 |
|  | White blood cell | Red blood cell | 0.24 | 0.17 | 61.5% | 1.36E-09 |
| **Stage 4 (De-acclimatization)** | LLS questionnaire | Circulation | 0.22 | 0.11 | 59.4% | 1.07E-04 |
|  | Red blood cell | Platelet | 0.56 | 0.29 | 95.3% | 1.60E-84 |
|  | White blood cell | Circulation | 0.29 | 0.16 | 66.9% | 9.77E-15 |
|  | White blood cell | Red blood cell | 0.27 | 0.18 | 65.0% | 1.39E-13 |
