# Supplementary material for: The composite phenotype analysis identifies potential concerted responses of physiological systems to high altitude exposure
Source: Natl Sci Rev. 2023 Mar 1;10(5):nwad053. doi: 10.1093/nsr/nwad053 (PMC10089582; doi:10.1093/nsr/nwad053)
Supplement: nwad053_Supplemental_Files [file nwad053_supplemental_files.zip › Supplementary-Table_S3.docx]

**Table S3.** **Canonical correlation summary of phenotypic network in different sample sets.**

| **Sampling 400** | **From** | **To** | **Max** | **Average** | **Stability** | **Pvalue** |
| --- | --- | --- | --- | --- | --- | --- |
|  |  |  | **CCA Rho** | **CCA Rho** |  |  |
| **Dynamic (4 time points)** | Kidney | Circulation | 0.34 | 0.20 | 78.79% | 2.69E-02 |
|  | Liver | Kidney | 0.73 | 0.32 | 99.47% | 1.92E-33 |
|  | LLS Questionnaire | Circulation | 0.45 | 0.24 | 92.07% | 4.85E-04 |
|  | Platelet | Liver | 0.46 | 0.25 | 93.75% | 5.56E-06 |
|  | Red Blood Cell | Circulation | 0.46 | 0.24 | 93.61% | 4.51E-05 |
|  | Red Blood Cell | Platelet | 0.88 | 0.39 | 99.89% | 7.57E-169 |
|  | Temperature | Red Blood Cell | 0.33 | 0.25 | 76.59% | 7.05E-02 |
|  | White Blood Cell | Circulation | 0.43 | 0.20 | 87.96% | 1.47E-05 |
|  | White Blood Cell | Platelet | 0.43 | 0.21 | 92.34% | 2.94E-06 |
|  | White Blood Cell | Red Blood Cell | 0.54 | 0.29 | 97.02% | 3.14E-17 |
| **Stage 1 (Baseline)** | Kidney | Circulation | 0.16 | 0.13 | 78.25% | 1.71E-04 |
|  | Liver | Kidney | 0.70 | 0.39 | 99.35% | 8.80E-111 |
|  | Platelet | Circulation | 0.19 | 0.09 | 78.85% | 2.80E-04 |
|  | Red Blood Cell | Circulation | 0.27 | 0.14 | 82.45% | 3.65E-09 |
|  | Red Blood Cell | Platelet | 0.86 | 0.35 | 99.86% | 2.38E-245 |
|  | White Blood Cell | Platelet | 0.31 | 0.16 | 84.68% | 9.85E-15 |
|  | White Blood Cell | Red Blood Cell | 0.30 | 0.16 | 84.13% | 1.42E-11 |
| **Stage 2 (Acute)** | Kidney | Circulation | 0.19 | 0.12 | 78.95% | 9.05E-05 |
|  | LLS Questionnaire | Circulation | 0.30 | 0.12 | 83.82% | 1.11E-09 |
|  | LLS Questionnaire | Kidney | 0.18 | 0.13 | 78.73% | 7.79E-04 |
|  | Red Blood Cell | Platelet | 0.48 | 0.25 | 94.60% | 2.24E-50 |
|  | Temperature | Red Blood Cell | 0.15 | 0.15 | 77.86% | 6.90E-03 |
|  | White Blood Cell | Platelet | 0.26 | 0.13 | 81.70% | 1.74E-09 |
|  | White Blood Cell | Red Blood Cell | 0.38 | 0.24 | 89.32% | 1.05E-29 |
| **Stage 3 (Chronic)** | Liver | Kidney | 0.27 | 0.18 | 82.60% | 6.95E-10 |
|  | Platelet | Liver | 0.27 | 0.13 | 82.15% | 1.46E-08 |
|  | Red Blood Cell | Liver | 0.24 | 0.09 | 80.68% | 5.73E-04 |
|  | Red Blood Cell | Platelet | 0.52 | 0.28 | 96.29% | 1.03E-69 |
|  | White Blood Cell | Circulation | 0.19 | 0.09 | 78.90% | 6.01E-04 |
|  | White Blood Cell | Red Blood Cell | 0.24 | 0.17 | 81.21% | 1.36E-09 |
| **Stage 4 (De-acclimatization)** | LLS Questionnaire | Circulation | 0.22 | 0.11 | 80.03% | 1.07E-04 |
|  | Red Blood Cell | Platelet | 0.56 | 0.29 | 97.35% | 1.60E-84 |
|  | White Blood Cell | Circulation | 0.29 | 0.16 | 83.70% | 9.77E-15 |
|  | White Blood Cell | Red Blood Cell | 0.27 | 0.18 | 82.92% | 1.39E-13 |
|  |  |  |  |  |  |  |
|  |  |  |  |  |  |  |
| **Sampling 200** | **From** | **To** | **Max** | **Average** | **Stability** | **Pvalue** |
|  |  |  | **CCA Rho** | **CCA Rho** |  |  |
| **Dynamic (4 time points)** | Kidney | Circulation | 0.45 | 0.28 | 78.53% | 4.97E-02 |
|  | Liver | Kidney | 0.76 | 0.40 | 98.85% | 1.23E-14 |
|  | LLS Questionnaire | Circulation | 0.59 | 0.33 | 94.59% | 3.37E-04 |
|  | Platelet | Liver | 0.61 | 0.34 | 96.05% | 2.64E-05 |
|  | Red Blood Cell | Circulation | 0.60 | 0.33 | 95.30% | 6.56E-04 |
|  | Red Blood Cell | Platelet | 0.90 | 0.45 | 99.58% | 1.76E-76 |
|  | Temperature | Red Blood Cell | 0.45 | 0.35 | 78.32% | 8.28E-02 |
|  | White Blood Cell | Circulation | 0.54 | 0.27 | 84.19% | 4.21E-04 |
|  | White Blood Cell | Platelet | 0.54 | 0.28 | 92.75% | 2.96E-04 |
|  | White Blood Cell | Red Blood Cell | 0.64 | 0.37 | 97.18% | 3.03E-09 |
| **Stage 1 (Baseline)** | Kidney | Circulation | 0.16 | 0.13 | 79.12% | 1.71E-04 |
|  | Liver | Kidney | 0.70 | 0.39 | 98.28% | 8.80E-111 |
|  | Platelet | Circulation | 0.19 | 0.09 | 79.15% | 2.80E-04 |
|  | Red Blood Cell | Circulation | 0.27 | 0.14 | 81.98% | 3.65E-09 |
|  | Red Blood Cell | Platelet | 0.86 | 0.35 | 99.23% | 2.38E-245 |
|  | White Blood Cell | Platelet | 0.31 | 0.16 | 83.65% | 9.85E-15 |
|  | White Blood Cell | Red Blood Cell | 0.30 | 0.16 | 83.32% | 1.42E-11 |
| **Stage 2 (Acute)** | Kidney | Circulation | 0.19 | 0.12 | 79.52% | 9.05E-05 |
|  | LLS Questionnaire | Circulation | 0.30 | 0.12 | 82.61% | 1.11E-09 |
|  | LLS Questionnaire | Kidney | 0.18 | 0.13 | 79.51% | 7.79E-04 |
|  | Red Blood Cell | Platelet | 0.48 | 0.25 | 91.88% | 2.24E-50 |
|  | Temperature | Red Blood Cell | 0.15 | 0.15 | 78.97% | 6.90E-03 |
|  | White Blood Cell | Platelet | 0.26 | 0.13 | 81.42% | 1.74E-09 |
|  | White Blood Cell | Red Blood Cell | 0.38 | 0.24 | 87.91% | 1.05E-29 |
| **Stage 3 (Chronic)** | Liver | Kidney | 0.27 | 0.18 | 82.71% | 6.95E-10 |
|  | Platelet | Liver | 0.27 | 0.13 | 81.72% | 1.46E-08 |
|  | Red Blood Cell | Liver | 0.24 | 0.09 | 80.26% | 5.73E-04 |
|  | Red Blood Cell | Platelet | 0.52 | 0.28 | 93.81% | 1.03E-69 |
|  | White Blood Cell | Circulation | 0.19 | 0.09 | 79.19% | 6.01E-04 |
|  | White Blood Cell | Red Blood Cell | 0.24 | 0.17 | 81.59% | 1.36E-09 |
| **Stage 4 (De-acclimatization)** | LLS Questionnaire | Circulation | 0.22 | 0.11 | 80.17% | 1.07E-04 |
|  | Red Blood Cell | Platelet | 0.56 | 0.29 | 94.97% | 1.60E-84 |
|  | White Blood Cell | Circulation | 0.29 | 0.16 | 83.01% | 9.77E-15 |
|  | White Blood Cell | Red Blood Cell | 0.27 | 0.18 | 82.94% | 1.39E-13 |
