# Supplementary figures and images for: The composite phenotype analysis identifies potential concerted responses of physiological systems to high altitude exposure
Source: Natl Sci Rev. 2023 Mar 1;10(5):nwad053. doi: 10.1093/nsr/nwad053 (PMC10089582; doi:10.1093/nsr/nwad053)

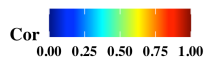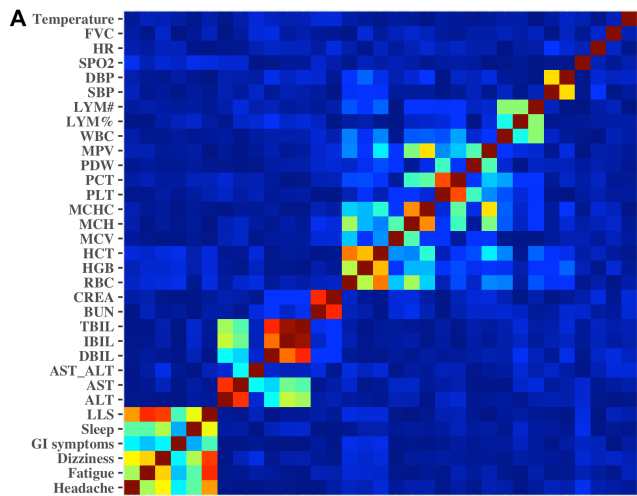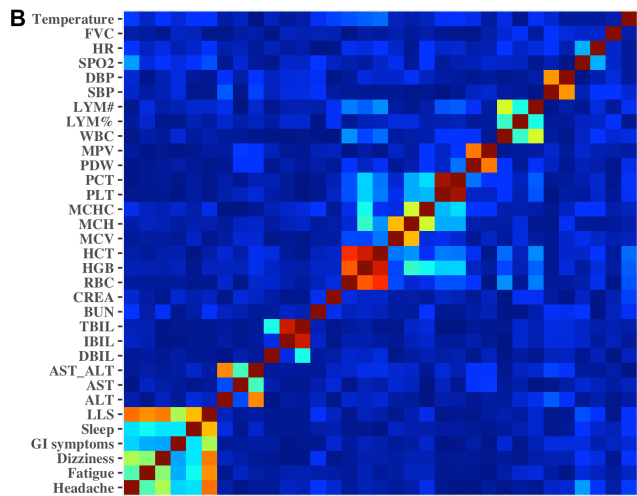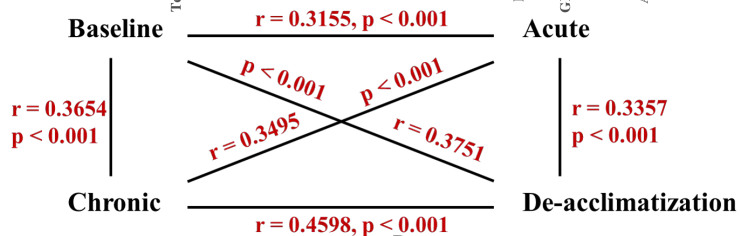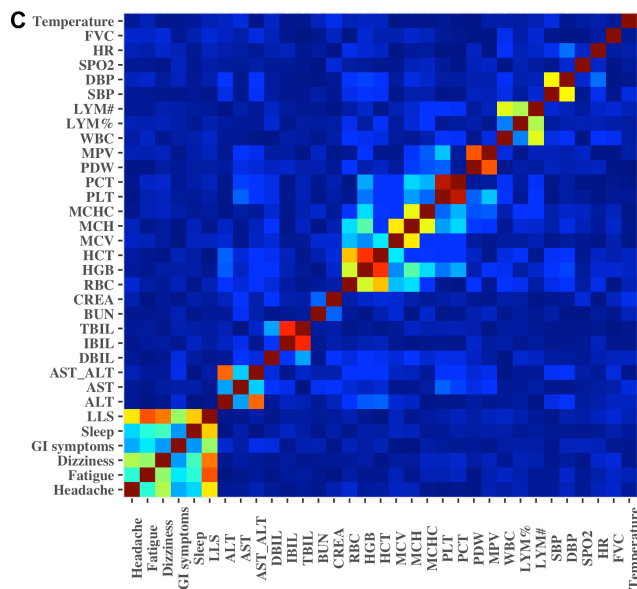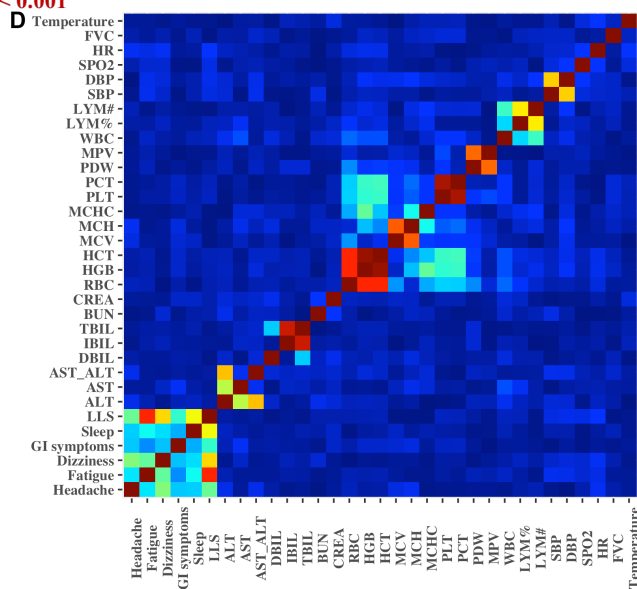

Supplement: nwad053_Supplemental_Files [file nwad053_supplemental_files.zip › Supplementary-Fig_S2.pdf]

A

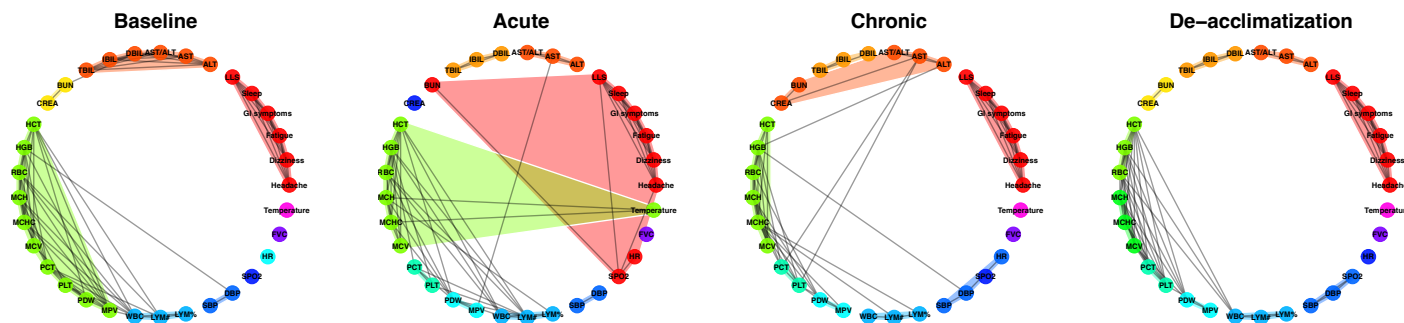

B

Clusters

Periods and Changes

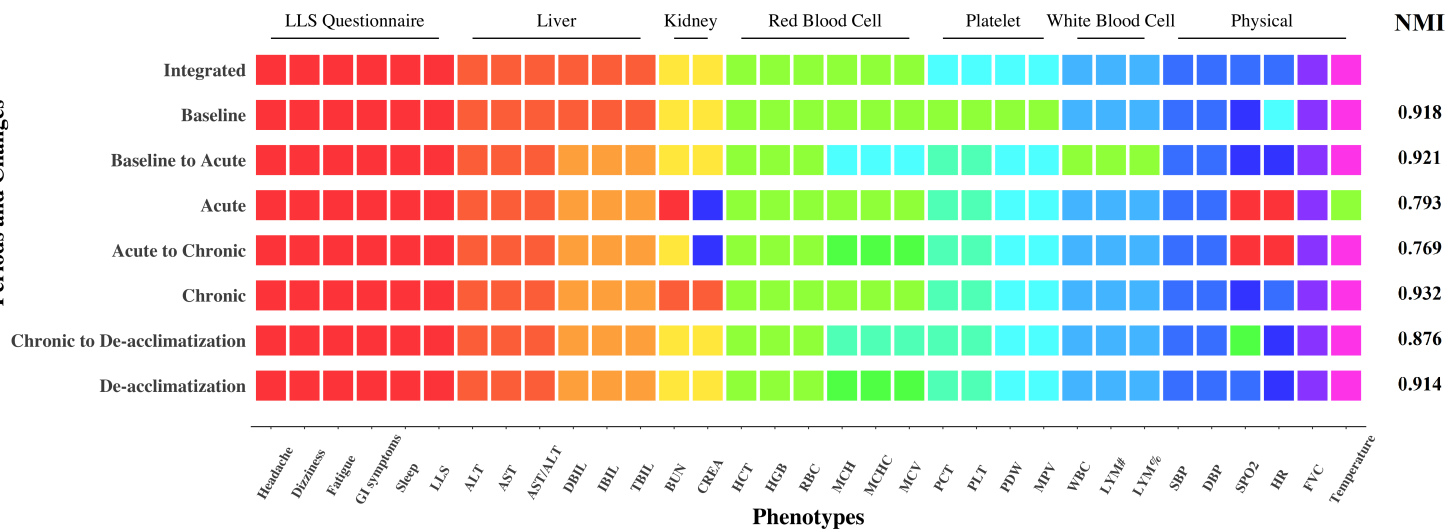

C

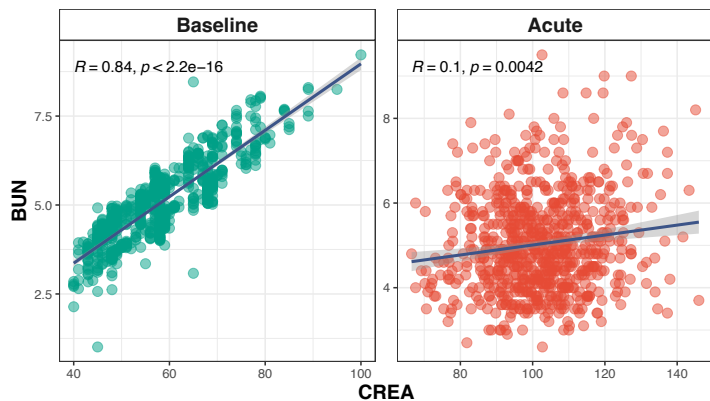

D

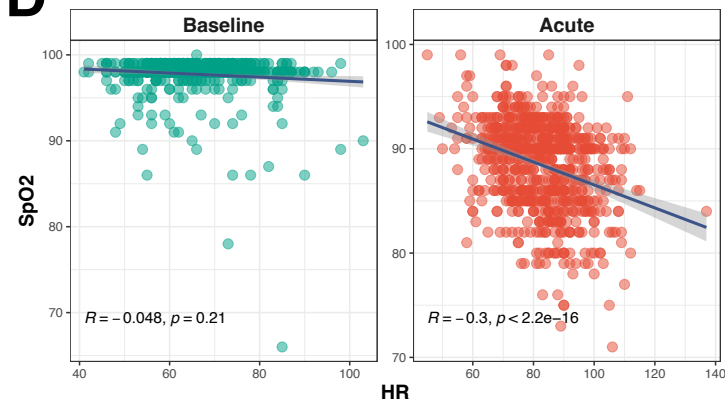

Supplement: nwad053_Supplemental_Files [file nwad053_supplemental_files.zip › Supplementary-Fig_S3.pdf]

A

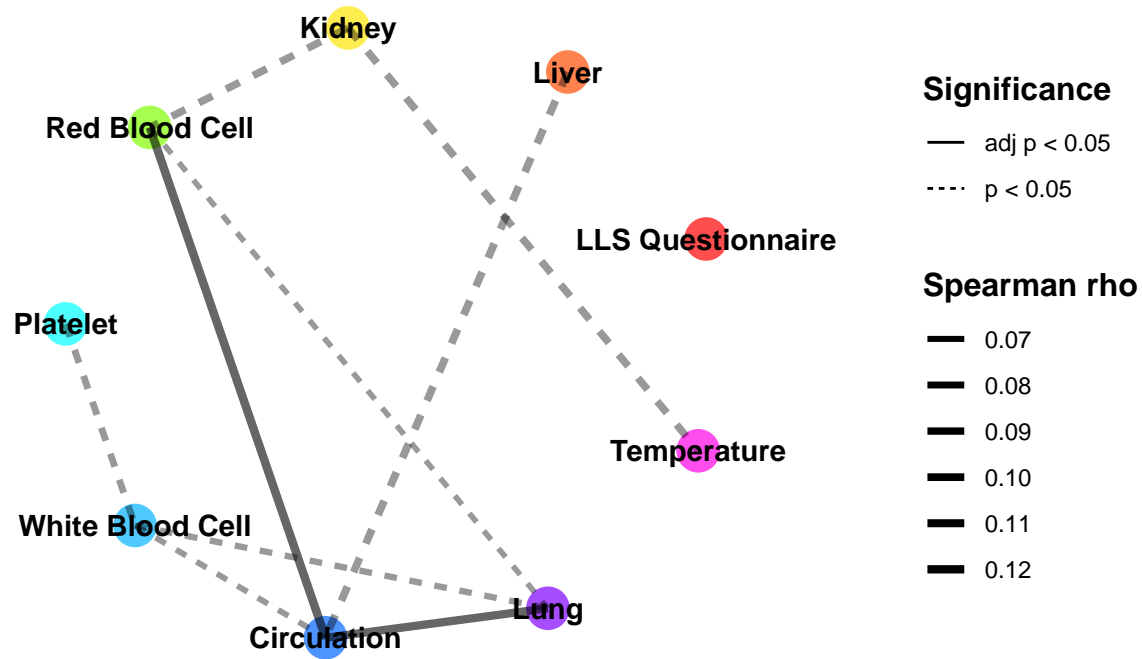

B

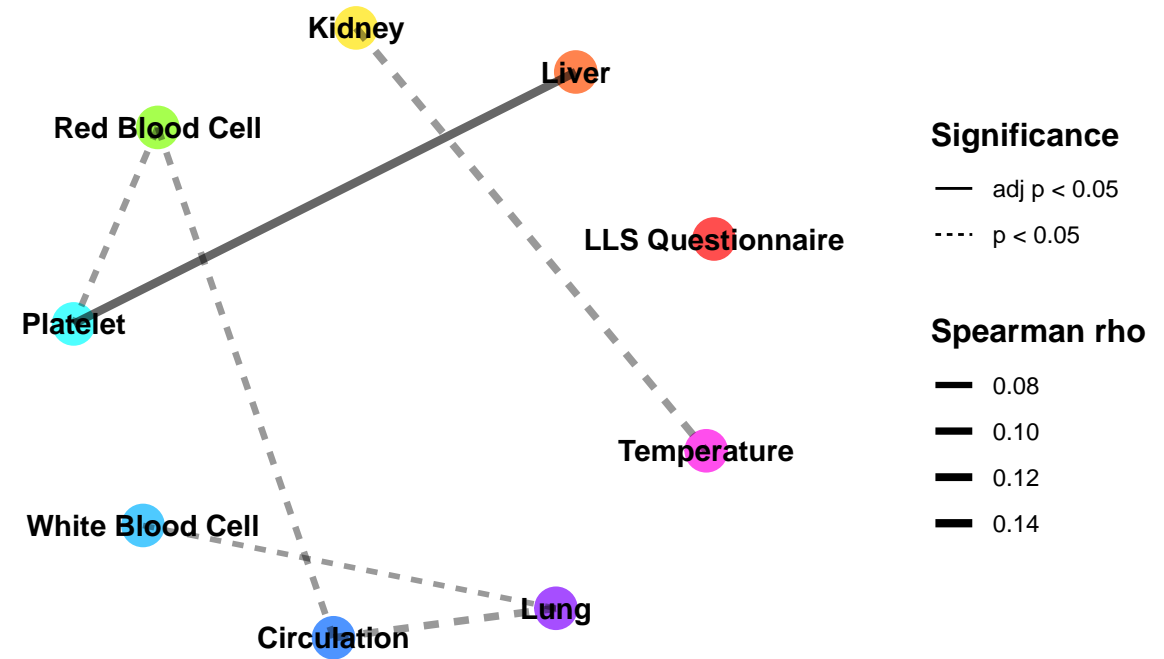

C

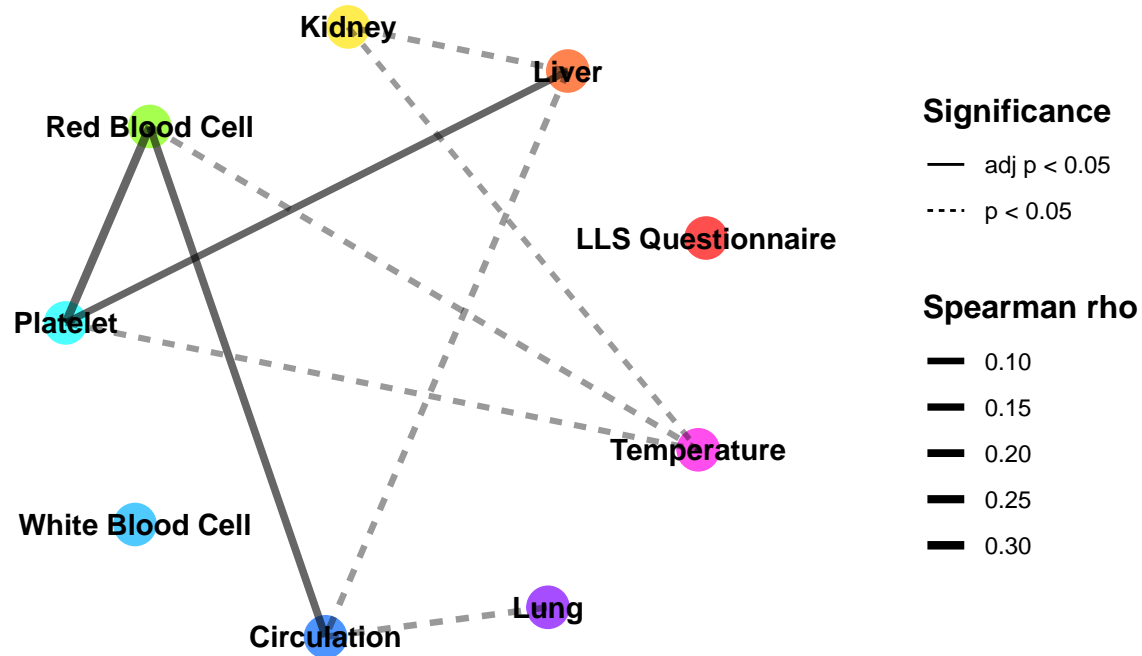

D

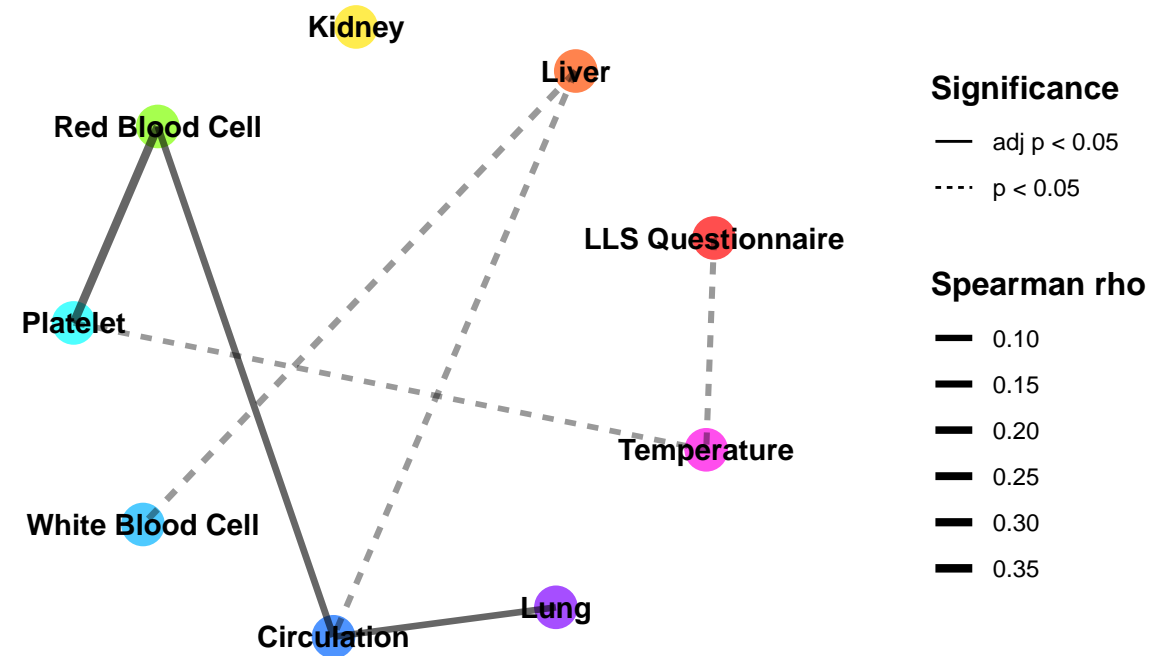

Supplement: nwad053_Supplemental_Files [file nwad053_supplemental_files.zip › Supplementary-Fig_S4.pdf]

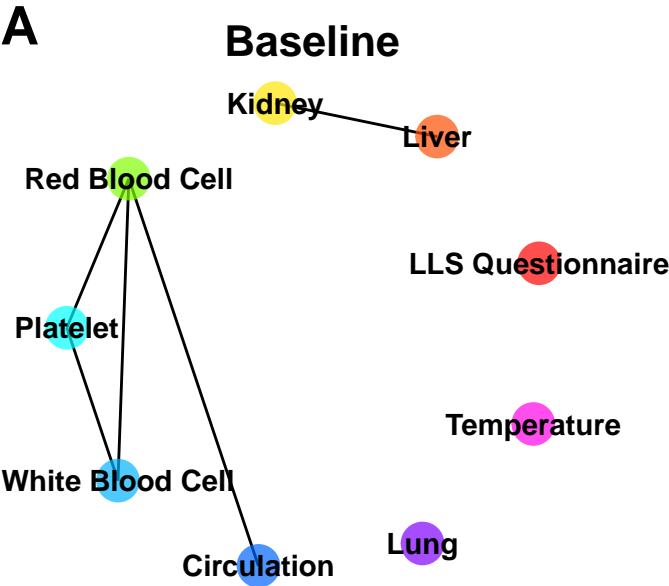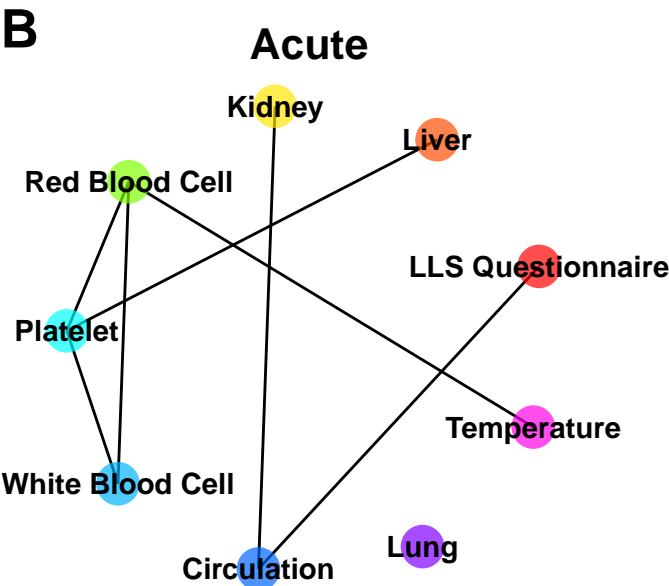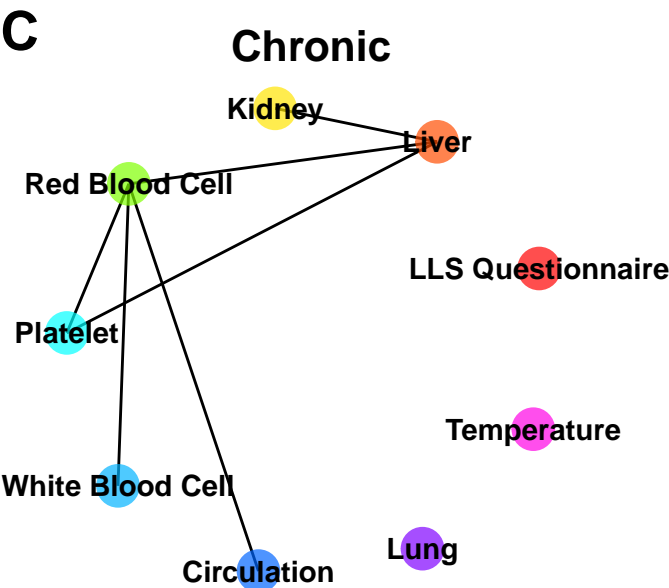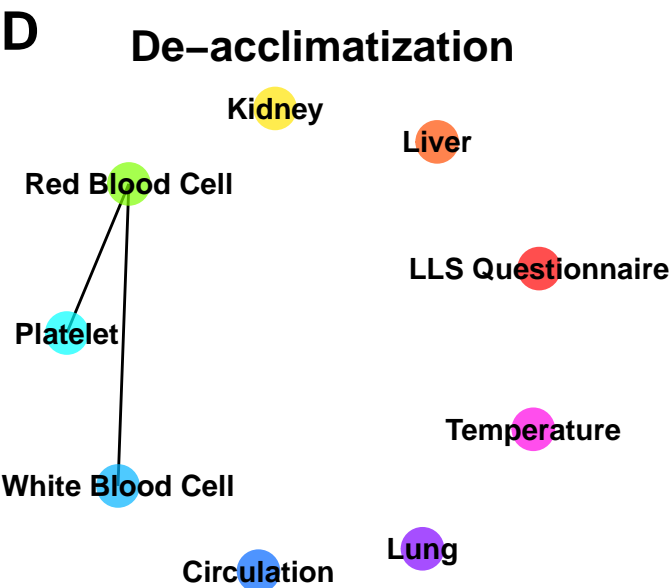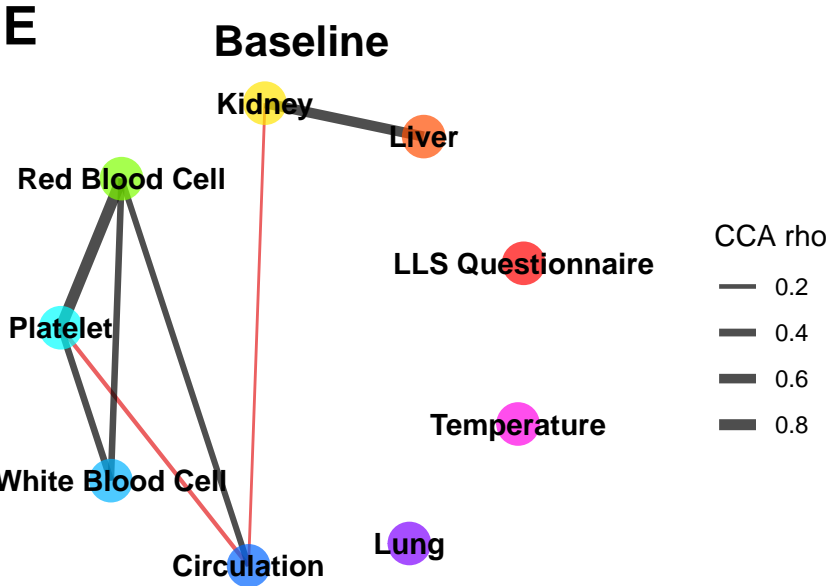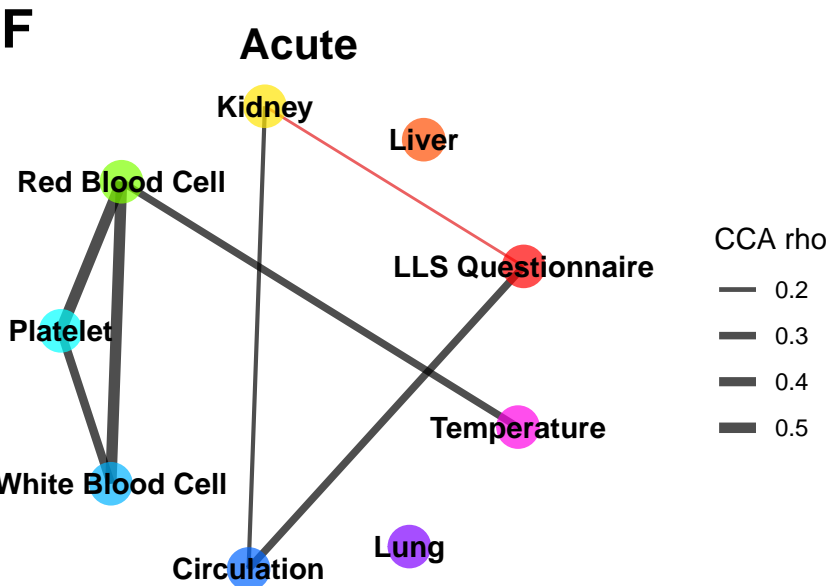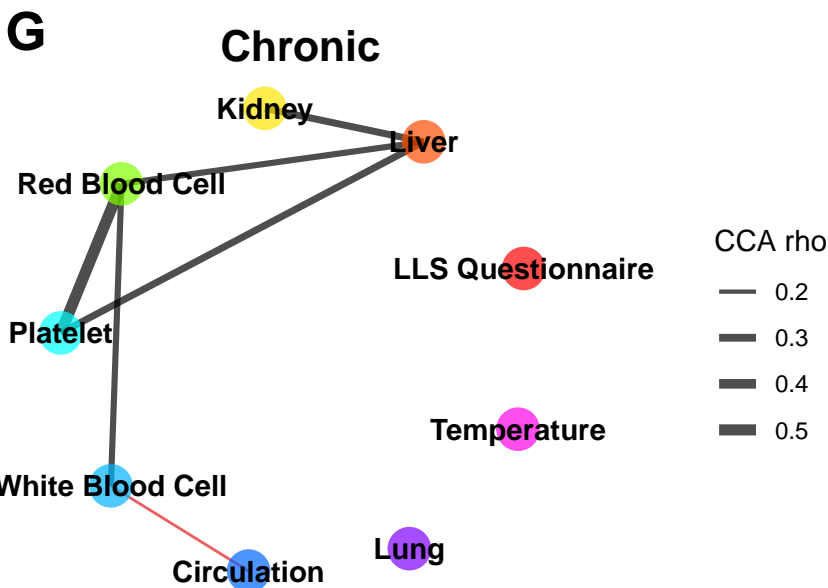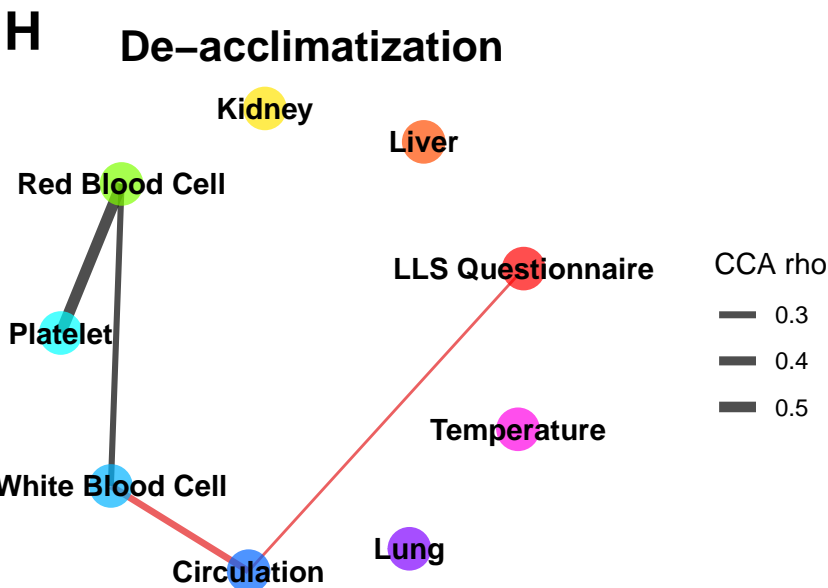

Supplement: nwad053_Supplemental_Files [file nwad053_supplemental_files.zip › Supplementary-Fig_S5.pdf]
